# Analysis of RNA conformation in endogenously assembled RNPs by icSHAPE

**DOI:** 10.1016/j.xpro.2021.100477

**Published:** 2021-04-28

**Authors:** Lu Chen, Howard Y. Chang, Steven E. Artandi

**Affiliations:** 1 Stanford Cancer Institute, Stanford University School of Medicine, Stanford, CA 94305, USA; 2 Department of Medicine, Stanford University School of Medicine, Stanford, CA 94305, USA; 3 Department of Biochemistry, Stanford University School of Medicine, Stanford, CA 94305, USA; 4 Center for Personal Dynamic Regulomes, Stanford University, Stanford, CA 94305, USA; 5 Howard Hughes Medical Institute, Stanford University, Stanford, CA 94305, USA

**Keywords:** Cell separation/fractionation, Sequencing, RNA-seq, Molecular Biology, Gene Expression, CRISPR, Protein expression and purification, Structural Biology

## Abstract

The majority of the mammalian genome is transcribed into non-coding RNAs, many of which co-evolve with RNA-binding proteins (RBPs) to function as biochemically defined and tractable ribonucleoproteins (RNPs). Here, we applied icSHAPE- a robust and versatile RNA structural probing pipeline- to endogenous RNPs purified from nuclei, providing base-resolution structural rationale for RNP activity and subcellular localization. Combining with genetic and biochemical reconstitutions, structural and functional alternations can be directly attributed to a given RBP without ambiguity.

For complete details on the use and execution of this protocol, please refer to Chen et al. (2018).

## Highlights

• Detergent-free extraction for native RNPs with high solubility and specific activity
• Affinity purify target RNPs to improve icSHAPE coverage and reproducibility
• CRISPR-KO and biochemical reconstitution to manipulate RBP’s occupancy
• Unambiguously assign structural footprints in the RNA to specific RBP subunits

## Graphical Abstract

**Figure:**
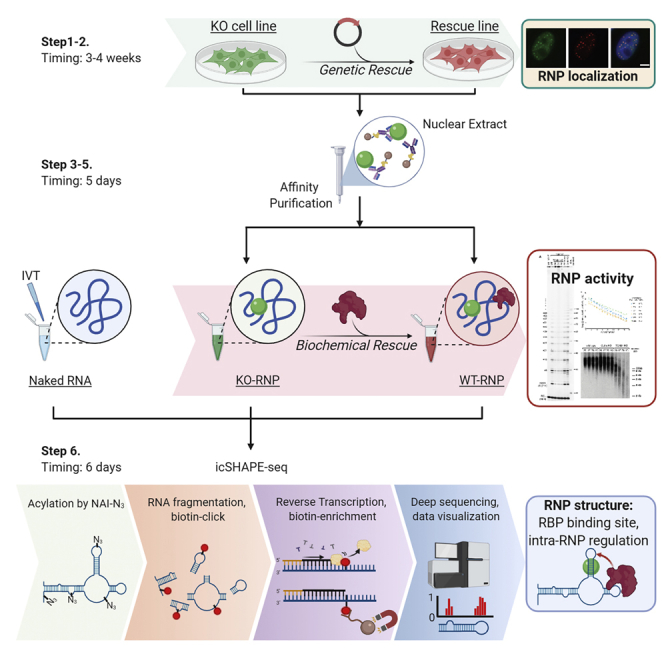

## Before you begin

The pervasiveness and wide-ranging abundance of ncRNAs have spurred debates on how their functionalities should be defined (Palazzo and Lee, 2015, Boland, 2017, Cech and Steitz, 2014). Needless to say, RNA structure provides the underlying basis for functions of ncRNAs/RNPs. Methods to capture the relevant RNP conformation in action during intracellular trafficking and catalysis are valuable to elucidate RNP regulations *in vivo* and *in vitro*. Here, we describe an experimental pipeline that enables direct coupling and cross-referencing of RNP’s cellular localization (step 1), assembly and catalytic activity (step 2) with RNA conformation captured by icSHAPE-seq (step 3).

icSHAPE (*in vivo* click selective 2-hydroxyl acylation and profiling experiments) was initially described to derive RNA secondary structure profile at a transcriptomic level by treating live cells with the icSHAPE modifier NAI-N_3_ (Flynn et al., 2016b, Flynn et al., 2016a, Spitale et al., 2015). We uniquely define how to adapt this versatile technique on endogenous assembled RNPs purified from distinct cellular contexts. Combining with genetic and biochemical manipulations of a specific RNP component, these approaches allow an unambiguous assignment of structural and functional changes in the RNP to a specific RNA binding protein (RBP) subunit.

**CRITICAL:** All steps should be carried out using RNase-free grade reagents and with standard practices to avoid RNase contamination. In addition, keep cells and all fractions of RNA/RNP/enzymes chilled throughout;snap-freeze and store any unused reagents in aliquots to minimize exposures in aqueous form and freeze-thawing cycles.

### In-house production of Cas9-sgRNA RNPs for gene deletion

**Timing: 6 h**

To interrogate an RBP’s contributions to RNP functions, we use loss-of-function approaches, such as siRNA-mediated knockdown (Chen et al., 2020a, Roake et al., 2019, Venteicher et al., 2009), and CRISPR-mediated gene inactivation (Chen et al., 2020a, Roake et al., 2019, Chen et al., 2018). For non-essential genes, CRISPR-mediated gene knockouts are preferred, because incomplete knockdown of the targeted gene can result in low level of the RBP proteinsufficient to sustain RNP functions, thereby masking RNP’s functional dependency on the RBP - in the case of TCAB1 (Chen et al., 2018, Venteicher et al., 2009). For essential genes, CRISPR-based inactivation tends to yield hypermorphs with only mild target reduction (Chen et al., 2020a, Chen et al., 2020b); whereas more efficient target depletion can be achieved using transiently introduced siRNAs (Chen et al., 2020b).

CRISPR editing can be achieved with ectopic expression of Cas9 and gRNAs from a plasmid, such as PX459 (Ran et al., 2013). Though the plasmid-borne method is widely used and user-friendly, we prefer to reconstitute the Cas9-gRNA RNP complex for these following reasons: firstly, promoters used to drive Cas9 cDNA and gDNA on the plasmid do not work for every target cells; secondly, plasmids can be integrated into the genome, causing prolonged Cas9 expression and cytotoxicity; thirdly, Cas9-sgRNA RNPs can readily edit genome upon cell entry without accumulating off-targeting edits due to cellular decay (Chen et al., 2016, Zuris et al., 2015). Here, we describe how to design and produce recombinant Cas9-sgRNA RNPs in-house.

**Figure 1:**
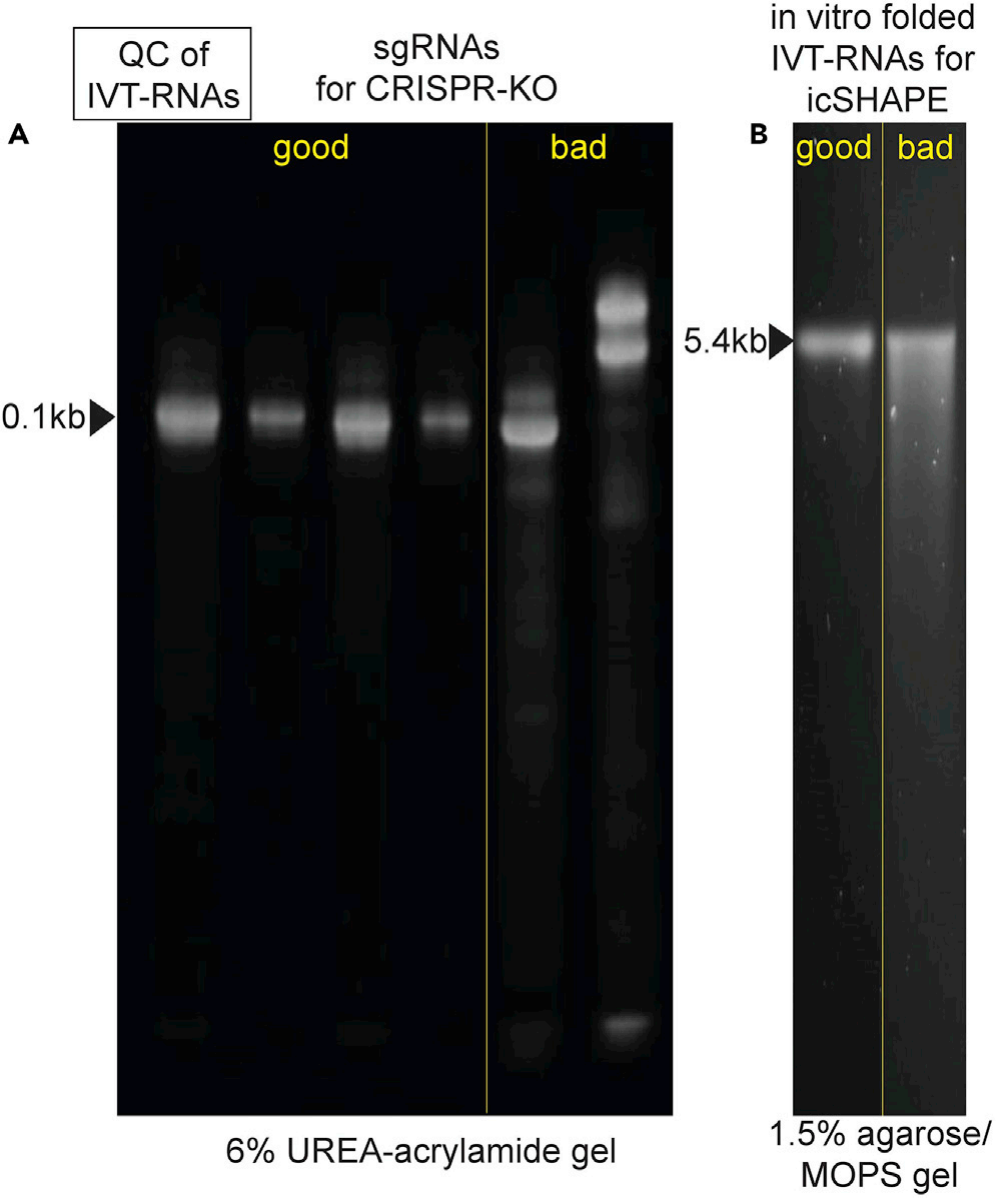
Quality control outcomes for RNAs produced by *in vitro* transcription “good” lanes exemplify optimal RNA preparations, where the majority of the products migrate at the molecular weight of the expected size (marked by black triangles); “bad” lanes show suboptimal reactions, where a significant portion of the products are either truncated or shorter than full length - comet tailing, potentially due to RNase contamination or incomplete elongation by the T7 polymerase. (A) 1 μg of IVT-sgRNAs (100nt) separated by a 6% UREA PAGE. (B) 1.5 μg of IVT-xCas9-ABE mRNA (5.4 kb) separated by a 1.5% agarose/MOPS gel.

1. Design and synthesis of sgRNAs by *in vitro* transcription (**IVT**)
  a. To engineer reliable loss-of-function alleles of a given RBP gene, we introduce genomic deletion at early exon(s) using a pair of CRISPR sgRNAs, which flank a genomic region up to 1kb.
    ***Note:*** Ideally, the flanked region should be exonic sequences of a high degree of conservation and/or functionality, such as an RNA binding motif or a catalytic domain. The design of sgRNAs can be facilitated by online tools such as CRISPOR/Tefor (Concordet and Haeussler, 2018).
  b. To enable IVT of sgRNAs by T7 RNA polymerase, order two overlapping DNA oligos as described in (Bassett et al., 2013), see materials and equipment for sequence details.
  c. Perform an overlapping PCR reaction to generate a dsDNA template for IVT.
    i. We use the Phusion PCR enzyme and the HF-buffer in a 100 μL reaction, see materials and equipment for the recipe and the PCR program.
    ii. Run an analytic gel for QC: if the PCR product does not appear predominantly as 123 bp, optimize the PCR reaction to maximize full-length yield.
    iii. Resuspend the bulk reaction using 5× volume of the PNI buffer (from the Qiagen PCR purification kit) and purify with a mini-Elute column.
      ***Note:*** To obtain the full-length PCR product with high yield, We tend to avoid gel extraction procedure for PCR-cleanup, as certain carry-overs using the commercial gel purification kit appear to interfere with IVT reactions.
    iv. Elute purified dsDNA templates in 10 μL of ddH_2_O.
      **CRITICAL:** High concentration and purity of the eluted dsDNA are the keys to an optimal IVT reaction. Use cautions during the column washing step to avoid introducing any carryovers.
  d. *In vitro* transcription (IVT) of sgRNAs using T7 polymerase.
    i. Set up a 20 μL reaction in PCR tubes at an ambient temperature around 22°C using the T7 high yield synthesis kit, with the maximum volume of dsDNA template included (see materials and equipment).
    ii. Incubate the reaction in a PCR machine at 37°C, do not exceed 2 h.
      ***Note:*** The reaction solution may become turbid which usually indicates high RNA yield.
    iii. Spike in 1 μL of turbo DNase, continue the 37°C incubation for 15min.
    iv. Purify the sgRNA using an RNA spin column.
      ***Alternatives:*** we purified high-quality RNAs with either the Monarch RNA Cleanup Kit or standard Trizol extraction.
    v. For QC of RNA integrity, mix 1 μg of the product with an equal volume of RNA loading dye (recipe in materials and equipment), heat at 75°C for 5min, and resolve in 7% Urea-PAGE gel (See materials and equipment), followed by SYBR-Gold staining.
      ***Note:*** The final sgRNAs are expected to run as a 100 nt band without apparent degradation. See Figure 1 and troubleshooting 1.

**Pause point:** Snap freeze aliquots of sgRNAs; store in −80°C.

2. Reconstitute Cas9-sgRNA RNPs
  a. Dilute the Cas9 protein in the *Cas9 working buffer* (see materials and equipment), mix in equal molar of sgRNAs.
  b. Incubate at 37°C for 5min to allow the Cas9-sgRNA RNP to assemble. Use immediately for RNP transfection into target cell lines in Major step 1-b.

### Generation of an antibody for RNP purification

**Timing: 6 months**

Affinity purification of RNPs greatly enriches the resident ncRNA, allowing *ex vivo* probing by icSHAPE with improved resolution (Major step 3), and enabling *in vitro* functional characterizations and biochemical reconstitution with recombinant RBPs (Major step 2). To this end, a high-quality antibody allows enrichment of endogenous RNPs from unedited cells, avoiding epitope-tagging or ectopic expression, both of which can alter the structural and functional properties of the RNP (Chiba et al., 2017, Stern et al., 2012). Affinity-purified, IP-grade antibodies for endogenous RNPs are not always available from commercial vendors. Whether an antibody can quantitatively enrich a specific RNP activity or components should be empirically determined, as shown in Figure 3 and in (Cohen and Reddel, 2008).

3. Choose an antigenic region(s) of RBP as an antigen:
  a. For low yield expressers, select a 70-80 amino acid region with high antigenicity score, high surface probability, and hign density of predicted turns.
  b. For high yield expressers, express the full-length protein as antigen.
4. Clone selected antigenic regions into two bacterial expression vectors,
  a. for antigen production, pMCH vector with an MBP tag.
  b. for affinity purification from serums, pGCH vector with a GST tag.
5. Express the MBP fusion protein in BL21 (DE3) competent cells by induction with 0.3 mM IPTG for 4 h at 30°C.
6. Purify the MBP fusion protein with standard amylose affinity chromatography, and elute by competitive maltose elution, according to the manual of NEB (E8021S) and (Bonhoure et al., 2018).
7. Immunize a host animal such as a rabbit or sheep with the purified antigen for polyclonal antibody production.
8. To prepare the affinity resin for antibody purification from serums.
  a. express the corresponding GST-tagged antigen in BL21(DE3) cells.
  b. Pass antigen-expressing lysates through an affinity column packed with glutathione resin, onto which the antigen is immobilized and crosslinked, using 14 mM DSS in DMF, rotating at 30°C for 45 min.
  c. Wash the affinity resin by gravity flow with 10× Column-Volume (CV) of 50 mM of *Glutathione (GSH) blocking buffer* (see materials and equipment) at an ambient temperature around 22°C.
  d. Wash by gravity flow with 5× CV of 0.1 M Glycine (pH 2.5) solution, followed by neutralization with 10× CV of 0.1 M Tris-HCl (pH 8.0) at an ambient temperature around 22°C.

**Pause point:** Store the sealed resin in 4°C for up to a year.

***Note:*** For QC purpose, set aside a small aliquot from each of these fractions: Input, flowthrough lysates, resins prior to DSS, and resins post-glycine wash. Assess the abundance of the antigen by anti-GST western blotting or Coomassie Blue staining.

9. To affinity-purify antibody from a crude serum:
  a. Load 5× CV of serum onto the affinity column, gentle rotating 10–14 h at 4°C.
  b. Wash the resin with 50× CV of PBS.
  c. Sequential elute the bound antibody with 12× 50% CV of 0.1M Glycine (pH 2.5), each into individual tubes containing 5% CV of 1 M Tris-HCl (pH 8.0). Neutralize by gently inverting the capped tube immediately after each elution.
    **CRITICAL:** minimize the exposure of the eluted antibody with the acidic glycine.
  d. Assess eluates for IgG by western blotting or Coomassie Blue staining.
  e. Pool peak fractions.
    ***Optional:*** Concentrate the peak fractions through a centrifugal filter device (molecular weight cutoff of 10 kDa).
    ***Note:*** the integrity of the affinity-purified IgG can be assessed by Coomassie blue staining, and by quantitative immune-depletion of a specific RNP activity shown in Figure 3.

### *In vitro* transcription to synthesize naked RNAs

**Timing: 5 h**

The structural profiling of a naked RNA by icSHAPE provides an important reference point for the default RNA folding state. When compared with the profile in its RNP context, base-resolution footprints of an RBP can be identified unbiasedly. Heredescribes the procedure we used to synthesize hTR - the noncoding RNA subunit of the human telomerase.

10. Clone hTR (451 nt) into a T7 promoter-containing vector- pCMV-T7-hTR (Fu and Collins, 2003).
11. Linearize the plasmid with EcoR1, which cuts right at the end of the hTR sequence. Digest 2 μg of plasmid DNA for 10–14 h with 5 μL EcoR1-HF in a 50 μL restriction digestion.
12. Resuspend the reaction thoroughly with 450 μL of PNI, and proceed with reaction cleanup with a DNA spin column.

***Note:*** wash the bound DNA twice with the Qiagen column washing buffer PE. Avoid any carry-overs.

**Pause point:** Elute DNA in 30 μL of ddH_2_O.

13. IVT synthesis using the NEB T7 high yield kit, using all 30 μL of linearized DNA as the template. Follow similar instructions in Preparation-1-d
14. For RNA species < 0.5 kb, QC the final IVT product by UREA-TBE PAGE; for longer RNAs, using a 1.5% MOPS-agarose gel instead (see materials and equipment)

***Note:*** for good and bad QC examples, see Figure 1 and troubleshooting 1.

## Key resources table

**Table:** 

REAGENT or RESOURCE	SOURCE	IDENTIFIER
**Antibodies**		

Anti-hTERT rabbit polyclonal (T421)	(Venteicher et al., 2008) and this study	n/a
Anti-hTCAB1 rabbit polyclonal	(Venteicher et al., 2008)	n/a
Anti-PCNA (PC10) mouse mAb	Cell Signaling Technology	2586T

**Bacterial and virus strains**		

BL21 (DE3)	NEB	C2527I
One Shot™ Stbl3™ Chemically Competent E. coli	Thermo/Invitrogen	C737303

**Chemicals, peptides, and recombinant proteins**		

TURBO™ DNase	Thermo/Invitrogen	AM2238
OmniPur® Formamide, Deionized - CAS 75-12-7 - Calbiochem	Millipore/Sigma	4610-100ML
SYBR™ Gold Nucleic Acid Gel Stain	Thermo/Invitrogen	S11494
TrueCut™ Cas9 Protein v2	Thermo/Invitrogen	A36498
BioUltra Urea for molecular biology	Millipore/Sigma	51456
Acrylamide/Bis Solution, 19:1	BioRad	161-0154
Pierce™ Glutathione Agarose	Thermo	16100
Amylose resin	NEB	E8021S
DSS (disuccinimidyl suberate)	Thermo	21655
L-Glutathione reduced (L-GSH)	Millipore/Sigma	G4251
Imperial™ Protein Stain	Thermo	24615
Opti-MEM™ I Reduced Serum Medium	Thermo/Gibco	31985062
Lipofectamine 2000 Transfection Reagent	Thermo/Invitrogen	11668030
Lenti-X™ Concentrator	Takara	631231
Polybrene Infection/Transfection Reagent	Millipore/Sigma	TR-1003-G
RNaseOUT	Thermo	1077019
Protease Inhibitor Cocktail	Millipore/Sigma	P8340
Deoxyribonuclease I from bovine pancreas	Millipore/Sigma	DN25
Protein A–Agarose Fast Flow	Millipore/Sigma	P3476
RNase A, DNase and protease-free	Thermo	EN0531
TRIzol™ LS Reagent	Thermo/Invitrogen	10296028

**Critical commercial assays**		

Phusion® High-Fidelity DNA Polymerase	NEB	M0530S
QIAquick PCR Purification Kit	QIAGEN	28104
HiScribe™ T7 High Yield RNA Synthesis Kit	NEB	E2040s
Monarch® RNA Cleanup Kit	NEB	T2040L
Zero Blunt™ TOPO™ PCR Cloning Kit	Thermo/Invitrogen	450245
Poly(A)Purist™ MAG Kit	Thermo/Invitrogen	AM1922

**Deposited data**		

icSHAPE data	This study	GSE97486

**Experimental models: cell lines**		

HeLa S3	ATCC	CCL-2.2
C57BL/6 mouse embryonic stem cells	ATCC; cultured as in (Buecker et al., 2014)	SCRC-1002
293T	ATCC	CRL-3216

**Oligonucleotides**		

Primers for in-house sgRNA production, see materials and equipment	Adopted from (Bassett et al., 2013)	n/a

**Recombinant DNA**		

pCMV-T7-hTR	(Fu and Collins, 2003)	A gift from K. Collins
pBluescript-U1-hTR	This study	Addgene 167456
pcDNA-3xFLAG-hTERT	This study	Addgene 167457
pCDNA-3xFLAG-GFP	This study	Addgene 167458
pCDH-3F-TCAB1-puro^R^	This study	Addgene 167459
pMGIB-HA-TCAB1	This study	Addgene 167460
pCDH-3F-GFP-puro^R^	This study	Addgene 167463
pMDLg/pRRE (encoding Gag and Pol, 3^rd^ gen lentiviral packaging)	This study	n/a
pRSV-Rev (encoding Rev, 3^rd^ gen lentiviral packaging)	This study	n/a
pCMV-VSVG (encoding VSV-G)	This study	n/a
xCas9(3.7)-ABE(7.10)	(Hu et al., 2018)	Addgene 108382
pMBP-TERT421-493 (N-terminal MBP-PreX tag with C-terminal TEV-His6 tag)	This study	Addgene 167461
pGST-TERT421-493 (N-terminal GST-PreX tag with C-terminal TEV-His6 tag)	This study	Addgene 167462

**Other**		

Zymo-Spin IC Columns	Zymo Research	C1004-250
SE600X Chroma Deluxe Dual Cooled Vertical Protein Electrophoresis Unit	Hoefer	SE600x
Amicon® Ultra-4 Centrifugal Filter Unit, 10kDa	Millipore/Sigma	UFC801024
NUTATOR™ Mixer Model	TCS Scientific	#117

## Materials and equipment

**Table:** Related to Preparation-1-b

In-house sgRNA synthesis	*5′ T7 promoter adapter (underscored)*	*sgRNA sequence*	*3′ overlapping adapter (in Bold)*
sgRNA-specific oligo	GAAATTAATAC GACTCACTATAG	*20nt*	**GTTTTAGAGC** **TAGAAATAGCAAG**
a second common oligo	AAAAGCACCGACTCGGT GCCACTTTTTCAAGTTG ATAACGGACTAGCCTTA TTTTAA**CTTGCTAT** **TTCTAGCTCTAAAAC**

**Table:** Related to Preparation-1-c-i

**100 μL PCR reaction**	**Final Concentration**	**Amount (μL) for 100 μL**
5x HF buffer	1x	20
dNTP mix 10 mM	250 μM	2.5
sgRNA-specific oligo 50 μM	0.5 μM	1
Common oligo 50 μM	0.5 μM	1
Phusion PCR enzyme	n/a	1
ddH_2_O	q.s. to 100 μL	74.5

**Table:** PCR cycling conditions_Preparation-1-c-i

**Steps**	**Temperature**	**Time**	**Cycles**
Initial Denaturation	98 °C	30 sec	1
Denaturation	98 °C	10 sec	35 cycles
Annealing	60 °C	30 sec	
Extension	72 °C	15 sec	
Final Extension	72 °C	5 min	1
Hold	4 °C	forever

**Table:** Related to preparation-1-d-i

20 μL IVT reaction	Final concentration	Amount for 20 μL
10× NEB T7 buffer	1×	2 μL
ATP 100 mM	10 mM	2 μL
GTP 100 mM	10 mM	2 μL
UTP 100 mM	10 mM	2 μL
CTP 100 mM	10 mM	2 μL
Linearized plasmid template or PCR product in ddH_2_O	q.s. 20 μL with maximum volume	8 μL
T7 RNA polymerase mix	1×	2 μL

***Note:*** set up the reaction at an ambient temperature around 22°C.

**Table:** Related to preparation-1-d-v

RNA PAGE loading dye	Stock concentration	Final concentration	Add to 10 mL
Deionized formamide	>99%	~ 95%	q.s. to 10 mL
Bromophenol blue	1% (w/v)	0.025% (w/v)	250 μL
Xylene cayanol FF	1% (w/v)	0.025% (w/v)	250 μL
EDTA (pH 8.0)	500 mM	5 mM	100 μL
SDS	10% (w/v)	0.025% (w/v)	25 μL

***Note:*** Aliquot the deionized formamide immediately after the bottle-opening. Snap-freeze aliquots in Liquid N2 or dry ice, and store in −20°C for up to 3 years.

**CRITICAL:** Do not autoclave the SDS stock solution; instead, pass through a 0.45 μM PES filter unit.

**Table:** Related to preparation-1-d-v

7% UREA-PAGE gel	Stock concentration	Final concentration	Add to 40 mL
BioUltra Urea powder	60.1 g/mol	8 M	19.2 g
TBE stock buffer	10×	1×	4 mL
Acrylamide/Bis solution, 19:1	30%	7%	9.3 mL
ddH_2_O	n/a	n/a	q.s. to 40 mL
*Dissolve these ingredients by rotating at 37°C; add the following immediately before gel-casting*			
Ammonium Persulphate (APS)	10% (w/v)	0.1% (w/v)	400 μL
TEMED	>99%	0.1% (v/v)	40 μL

**CRITICAL:** do not heat the urea solution, as it speeds up urea’s breakdown.

***Optional:*** Urea-TBE-acrylamide/Bis stock solution with the desired acrylamide concentration can be made in a larger batch, and stored in 4°C for up to 6 months; Protected from light.

**Table:** Related to preparation-2-a

Cas9 working buffer	Stock concentration	Final concentration	Add to 10 mL
HEPES-NaOH (pH 7.9)	1000 mM	20 mM	200 μL
KCl	1000 mM	150 mM	1.5 mL
ddH_2_O	n/a	n/a	8.3 mL

***Note:*** do not autoclave the HEPES stock solution; instead, pass through a 0.22 μM PES filter unit. We store aliquoted solution in −20°C for up to 3 years.

**Table:** Related to preparation-8-c

GSH blocking buffer	Stock concentration	Final concentration	Add to 50 mL
Tris-HCl (pH 8.0)	1 M	0.1 M	5 mL
NaCl	5 M	150 mM	1.5 mL
ddH_2_O	n/a	n/a	q.s. 50 mL
L-GSH powder	307 g/mol	50 mM	767.5 mg

***Note:*** we store aliquoted solution in −20°C for up to 3 years.

**Table:** Related to preparation-15

1.5% agarose-MOPS gel casting	Stock concentration	Final concentration	Add to 100 mL
Agarose powder	n/a	1.5% (w/v)	1.5 g
ddH_2_O	n/a	n/a	72 mL
Boil it in a microwave oven, cool down to about 55°C.			
10× MOPS stock buffer (see below)	10×	1×	10 mL
Formaldehyde	12 M	2.2 M	18 mL

***Note:*** 1.5% agarose-MOPS gel is capable of resolving RNA from 0.5–8 kb.

**CRITICAL:** add the formaldehyde solution in a fume hood.

**Table:** Related to preparation-15 (continued)

10× MOPS stock buffer	Stock concentration	Final concentration	Add to 1000 mL
MOPS powder	n/a	0.2 M	41.8 g
Dissolve the MOPS powder in 700 mL of ddH_2_O, adjust pH to 7.0 with a few mL of 10 N NaOH			
Sodium acetate (pH=5.2)	3 M	20 mM	6.7 mL
EDTA (pH=8.0)	0.5 M	10 mM	20 mL
ddH_2_O	n/a	n/a	q.s. to 1000 mL

***Note:*** Pass the solution through a 0.45 μM PES filter unit. Store at an ambient temperature around 22°C for at least a year, protected from light.

**Table:** Related to Major Step-3-b

Buffer A	Stock concentration	Final concentration	Add to 1000 mL
HEPES-NaOH (pH 7.9)	1 M	10 mM	10 mL
MgCl_2_	1 M	1.5 mM	1.5 mL
KCl	1 M	10 mM	10 mL

***Note:*** Pass the solution through a 0.45 μM PES filter unit. Store at 4°C for at least a year.

**Table:** Related to Major Step-3-h

Buffer B	Stock concentration	Final concentration	Add to 1000 mL
HEPES-NaOH (pH 7.9)	1 M	300 mM	300 mL
MgCl_2_	1 M	30 mM	30 mL
KCl powder	75 g/mole	1400 mM	105 g
Add an equal volume of 100% glycerol and Buffer B to reconstitute the cytosolic extract – S100.

***Note:*** Pass the solution through a 0.45 μM PES filter unit. Store at 4°C for at least a year.

**Table:** Related to Major Step-3-i

Buffer C	Stock concentration	Final concentration	Add to 1000 mL
HEPES-NaOH (pH 7.9)	1 M	20 mM	20 mL
Glycerol	100%	25%	250 mL
MgCl_2_	1 M	1.5 mM	1.5 mL
EDTA	0.5M	0.2 mM	0.4 mL

***Note:*** Pass the solution through a 0.45 μM PES filter unit. Store at 4°C for at least a year.

**Table:** Related to Major Step-3-q

10× DNaseI buffer	Stock concentration	Final concentration	Add to 10 mL
Tris-HCl (pH=7.5)	1 M	100 mM	1 mL
MgCl_2_	1 M	25mM	0.25 mL
CaCl_2_	2.5 M	5 mM	20 μL
DNaseI powder	400 Kunitz Unit/mg	4000 Kunitz Unit/mL	100 mg
Glycerol	100%	10%	1 mL
ddH_2_O	n/a	n/a	q.s. 10mL

***Note:*** Pass the solution through a 0.45 μM PES filter unit. Store in −20°C for up to 3 years.

**Table:** Related to Major Step-4-c/d

Nuclear Buffer420 w/ BSA	Stock concentration	Final concentration	Add to 40 mL
PBS	n/a	n/a	20 mL
Buffer C	1×	n/a	16.6 mL
NaCl	5 M	420 mM	3.4 mL
BSA fraction-V powder		0.5 mg/mL	20 mg

***Note:*** BSA should be omitted for the BSA-free version.

***Note:*** Pass the solution through a 0.45 μM PES filter unit. Store in −20°C for up to 3 years.

**Table:** Related to Major Step-4-k

Lys450 buffer	Stock concentration	Final concentration	Add to 1000 mL
HEPES-KOH (pH=7.9)	1 M	20 mM	20 mL
NaCl	5 M	450 mM	90 mL
Triton X-100	100%	0.5%	5 mL
KCl	1 M	10 mM	10 mL
MgCl_2_	1 M	4 mM	4 mL
EDTA (pH=8.0)	0.5 M	0.2 mM	0.4 mL
Glycerol	100%	10%	100 mL
ddH_2_O	n/a	n/a	q.s. 1000 mL

***Note:*** Pass the solution through a 0.45 μM PES filter unit. Store in 4°C for at least 1 year.

**Table:** Related to Major Step-4-k

NP150 buffer	Stock concentration	Final concentration	Add to 1000 mL
HEPES-KOH (pH=7.9)	1 M	25 mM	25 mL
KCl powder	n/a	150 mM	11.18 g
MgCl_2_	1 M	1.5 mM	1.5 mL
NP-40	100%	0.5%	5 mL
Glycerol	100%	10%	100 mL
ddH_2_O	n/a	n/a	q.s. 1000 mL

***Note:*** Pass the solution through a 0.45 μM PES filter unit. Store in 4°C for at least 1 year.

**Table:** Related to Major Step-4-l

10× Direct assay buffer	Stock concentration	Final concentration	Add to 50 mL
Tris-HCl (pH = 8.0)	1 M	50 mM	25 mL
NaCl	5 M	500 mM	5 mL
MgCl_2_	1 M	10 mM	0.5 mL
Spermidine	1 M	10 mM	0.5 mL
β-mercaptoethanol	14.3 M	50 mM	0.175 mL
ddH_2_O	n/a	n/a	q.s. 50 mL
For 50mL 1× working solution w/ 30% glycerol: 5mL of 10× stock + 15 g glycerol, ddH_2_O q.s. 50 mL

***Note:*** Pass the solution through a 0.45 μM PES filter unit. Store in 4°C for at least 1 year.

***Note:*** weighing glycerol facilitates the preparation; the receipt was adopted from https://www.colorado.edu/lab/cech/lab-protocols and (Zaug et al., 2013)

## Step-by-step method details

### RBP knockout and genetic reconstitution in mammalian cells

**Timing: 3–4 weeks**

This Major Step describes the procedure to generate RBP-KO cell lines, from which wild type RNPs or RNPs deficient of a given RBP can be purified. We also describe how to genetically rescue these KO cells with the RBP cDNA. These cell lines can be used to dissect RNP’s cellular functions that depend on the contribution of a given RBP (Chen et al., 2018, Chen et al., 2020a, Chen et al., 2020b). Finally, genetically reconstituted cell lines expressing an epitope-tagged RBP provide a convenient source for recombinant RBPs to be used in the subsequent biochemical reconstitution (Major step 5).

1. Genomic targeting of the RBP by Cas9-sgRNA RNPs
  a. Seed 3 × 10^4^ HeLa or mouse embryonic stem cells (mESCs) in a 48-well plate one night before the transfection.
  b. Transfect reconstituted Cas9-sgRNA RNPs (20 pmol of Cas9 protein, and 20 pmol of sgRNA), together with 2 pmol of pcDNA-GFP, using 1.5 μL of Lipofectamine 2000 diluted in Opti-mem.
    ***Alternatives:*** plasmid-borne Cas9 and sgRNAs, e.g., PX459 (Ran et al., 2013), can also deliver genome-editing to cells by transfection, also see the discussion in Preparative step 1.
    ***Note:*** co-transfect with the GFP-expressing vector enables the enrichment of positive-transfectants by FACS-sorting.
  c. 3 days post-transfection, sort GFP-expressing single cells into two 96-plates.
    ***Alternatives:*** seed 1000 cells sparsely in a 10cm dish for cell cloning.
  d. Pick single HeLa colonies after about 14 days, and about 10 days for mESCs.
    **Pause point:** Prepare cells for colony screeening and freeze down a replicate plate.
    ***Note:*** No medium change is necessary during clonal growth.
  e. Screen for KO clones based on the RBP expression using western blotting or immunofluorescence.
  f. To analyze the genomic sequence of the targeted locus, design two PCR primer pairs,
    i. Inside amplicon for detecting unedited alleles: within the two sgRNA cutting sites.
    ii. Outside amplicon for detecting edited alleles: flanking the region to be deleted.
      ***Note:*** Successful homozygous deleted clones are expected to be negative for the inside amplicon, while positive for the outside amplicon.
  g. The outside amplicon can be further cloned into a TOPO vector, followed by colony sequencing to examine the exact genomic composition post-editing.
    ***Note:*** we routinely observe ∼25% clones with monoallelic deletions and ∼7% clones with biallelic deletions.

2. Genetic rescuing the KO cell line with an epitope-tagged cDNA of RBP.
  a. Clone cDNA of the RBP in-frame with an epitope tag, into a lentiviral packaging vector such as pCDH-CMV-puro.
    **CRITICAL:** since the pCDH contains long terminal repeats (LTRs) that are prone to recombination, we recommend propagating them in the Stbl3 stain with reduced recombination frequency.
  b. For lentivirus packaging, we transfect 293T cells seeded one-night prior in a 10-cm dish at 50% confluency.
  c. dilute the following 3^rd^ generation packaging plasmids in 1 mL Opti-MEM:
    i. pMDLg/pRRE: 4.5ug
    ii. pCMV-VSVG: 2.5ug
    iii. pRSV-Rev: 1.5ug
    iv. pCDH-tag-RBP-Puro^R^ or pCDH-GFP-Puro^R^ control: 7ug
  d. In a separate tube, resuspend 50 μL of lipofectamine in 1 mL of OptiMEM.
  e. Combine and mix contents from two tubes, incubate at an ambient temperature around 22°Cfor 20 min.
  f. Add the 2mL cocktail into 293T cells in Opti-mem, incubate for 6 h under standard tissue culture condition.
  g. Replace the dish with a complete culture medium.
  h. 48 h post-transfection, collect the 1^st^ batch of culture medium, and store it at 4°C; replenish the remaining cells with fresh medium.
    **CRITICAL:** protect the viral supernatant from lights, and handle it with biosafety level 2 precautions.
  i. 72 h post-transfection, combine both supernatants, pass through a 0.45 μM polyethersulfone filter using a syringe.
    **CRITICAL:** do not use the 0.22 uM filter, as virus particles may be damaged.
  j. To concentrate the viral supernatant, add 1/3 the volume of Lenti-X concentrator, incubate 10–14 h at 4°C. Protect it from light.
    ***Alternatives:*** the filtered unconcentrated viral supernatant can be directly used to infect cells.
  k. The next day, spin at 1500 × *g* for 45min at 4°C
  l. Resuspend the visible viral pellet in an 800 μL complete culture medium containing 10% FBS. This concentrated viral solution can be directly applied to infect target cell hosts.
    ***Alternatives:*** concentrated viral supernatants can be aliquoted, snap-frozen, and stored in −80°C for future infection. Note potential drop of viral titer can occur during freezing and thawing.
  m. To infect target host cells, seed 5 × 10^4^ cells per well of a 6-well plate with a complete medium containing 10 μg/mL polybrene, incubate for 1 h in the TC incubator.
  n. Swirl-in 100 μL of concentrated viral solution into the culture medium.
    ***Note:*** GFP expression in the GFP-transduced cells should be visible by 24 h, which can be used to track the progression of the transgene expression.
  o. 48 h post-infection or whenever most control cells become GFP-positive, begin antibiotic selection by adding 2 ng/μL puromycin.
    ***Note:*** We expect complete killing of the mock-transduced control cell within 2 days; while expecting pCDH-transduced cells to show minimum cell death, if at all. Minimum cell death indicates efficient viral transduction. These rescued cells can be further expanded in reduced puromycin.
    **CRITICAL:** To maintain stable expression of the transgene, avoid extensive passages; instead, go back to a low-passage stock.
  p. Instructions and examples for immunofluorescence, single-molecule RNA FISH, and biochemical assays can be found in (Chen et al., 2020a, Chen et al., 2018)

### RNP purification and biochemical reconstitution

**Timing: 5 days**

Efficient cellular fractionations can achieve a significant enrichment for RNPs. Cellular compartments and fractionation procedures may differ per RNP of interest. Here we describe a fractionation procedure relevant for multiple RNPs that differentially distribute between the cytosolic, nucleoplasm, and chromatin compartments. Specifically, we avoid detergent usage, minimize mechanical disruptions, and rely on high salt extraction; thereby, a pool of RNP complexes with high solubility, specific activity, and native folding states are preferentially extracted and enriched. The subsequent purification step depends on an affinity-purified, homemade antibody to purify endogenous RNP complexes with native stoichiometry and specific activity (Figure 3), avoiding potential complications introduced by epitope tagging or overexpression of exogenous constructs (Chiba et al., 2017, Stern et al., 2012).

Quality controls during extract preparation and RNP purification are crucial because introducing molecular aggregates and insolubles into the icSHAPE reaction can complicate data interpretation. For QC of RNP purification and production, assaying a specific activity of RNPs is preferred, as shown with the telomerase assay in Figure 3. If such an assay is not available, it is recommended to track the abundance of RNP components in solubilized fractions, e.g., quantitatively assessing protein or RNA levels in “Input extract”, “antibody-depleted extract”, and “eluate” using western and northern blottings.

**Figure 2:**
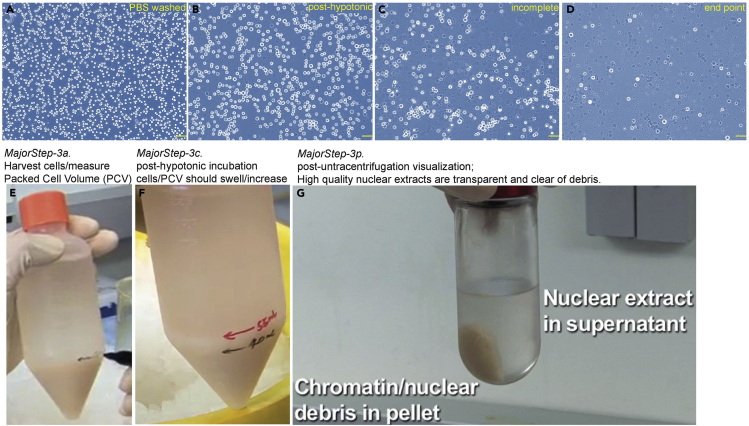
Key visualizations during the Dignam nuclear extraction in Major step 3 (A–D) 293T cells or their nuclei stained by 0.2% Trypan Blue. Intact cells, unstained bright dots; nuclei are stained as darker dots. Pictures were obtained with a 5× objective; yellow scale bars denote for 100 μmM. (E) measure packed cell volume (PCV) upon cell harvesting. (F) post-hypotonic inbuation, cells swell in volume. (G) post-ultracentrifugation visualization.
  b. Resuspend the packed cells in 5× PCV of hypotonic *Buffer A* (see materials and equipment) with gentle pipetting.
  c. Incubate the cell suspension on ice for an exact 10min to let cells swell.
  d. Spin down cells 250 × *g* for 5min at 4°C, and remove the supernatant.
    ***Note:*** as noted in Figure 2F, the PCV increased from 40mL to 55mL due to cell swelling, also see Figure 2B.
    **CRITICAL:** post-hypotonic cells become fragile, and pre-mature rupturing of cells is often observed (Figure 2B). The timing and gentle handling are keys to minimize rupturing prior to homogenization.
  e. Resuspend the pellet in 2× PCV of *Buffer A*, and transfer to a Dounce homogenizer.
  f. homogenize cell suspension with a LOOSE pestle, until 90% of cells are positively stained with Trypan Blue (0.2% final concentration) under a light microscope (see Figure 2C for an example of incomplete homogenization, and Figure 2D for the desired endpoint).
    **CRITICAL:** avoid over-homogenizing the nuclei, which compromises the quality of the final extract. The minimum necessary number of strokes for each cell type needs to be determined empirically (Figures 2C and 2D).
  g. Spin down the homogenates at 25,000 × *g* for 20min at 4°C.
  h. Transfer the supernatant, add 11.1% (v/v) of glycerol and 11.1% (v/v) of *Buffer B* (see materials and equipment) to reconstitute the final S100.
    ***Note:*** the salt concentration of the final S100 extract is ∼280 mM.
    ***Note:*** S100 contains soluble cytosolic molecules and a fraction of nuclear molecules that inevitably leak out.
    **CRITICAL:** Avoid collecting debris from the nuclei pellet; also avoid introducing any insoluble debris from the S100 supernatant; re-centrifuge the S100 to pellet any insoluble if necessary.
  i. Aspirate and discard any leftover supernatant with a pipette and add *Buffer C* (see materials and equipment) to the nuclei pellet, swirl gently to resuspend, and transfer to a homogenizer.
    ***Note:*** For every 1mL of starting PCV - an equivalent of ∼3 × 10^8^ mammalian cells, add 0.83mL of *Buffer C*.
  j. Homogenize nuclei by a couple of strokes using a LOOSE glass pestle.
    **CRITICAL:** avoid over-homogenizing the nuclei resuspension.
  k. Pour the homogenates into a chilled beaker.
    ***Note:*** choose a properly sized beaker to allow efficient mixing of the solution (see Methods video S1)
    Methods video S1. Key visuals for the Dignam nuclear extraction, related to Major step 3 Major Step-3k: transfer nuclei into a chilled beaker with appropriate size.
    Major Step-3l: drop-wise add 5M NaCl to the beaker while maintaining gentle stirring.
    Major Step-3m: the pellet becomes viscous and gooey; transfer to a centrifuge tube.
  l. To extract nuclear molecules with high salt, drop-wise apply 5M NaCl solution to the homogenate, while maintaining a gentle stirring motion (see Methods video S1). The final concentration of NaCl should be around 0.42 M.
    ***Note:*** use the equation below to calculate the volume of 5M NaCl needed:
    ***Note:*** with all the NaCl added, the suspension should turn viscous and gooey. (see Methods video S1).
  m. Pour the nuclei suspension into an ultracentrifugation tube, e.g. type Ti-45, with care.
  n. Gently rotate the tube for 30 min at 4°C.
  o. Ultracentrifuge at 40,000 × *g* for 30 min at 4°C.
  p. Aliquot and store the supernatant as the high salt nuclear extract.
    ***Note:*** a quality nuclear extract should be visually transparent without debris (Figure 2G). Harsh handling or over-homogenizing of cells or nuclei can introduce unwanted damages to the chromatin, resulting in insoluble debris that refrains from centrifugation. See troubleshooting 2.
  q. To release chromatin-bound molecules, rinse the pellet with PBS twice, and resuspend in a volume of *1x DNaseI digestion buffer* (400 Kunitz Unit/mL) that is proportional to the starting cell number.
    ***Note:*** use 200 Kunitz Units of DNase I per 1 × 10^7^ cells (for the recipe of a 10× stock buffer, see materials and equipment).
  r. Gently rotate for 30 min at 37°C
  s. Spin down 21,000 × *g*, 1min at RT to pellet any insoluble.
  t. Store the supernatant as the salt-resistant, DNase-sensitive chromatin-bound fraction.
    ***Note:*** for QC of the chromatin-bound extracts by western blotting, histones and other chromatin-associating factors such as PCNA should be relatively enriched; whereas, the β-tubulin signal should be relatively depleted.

**Figure 3:**
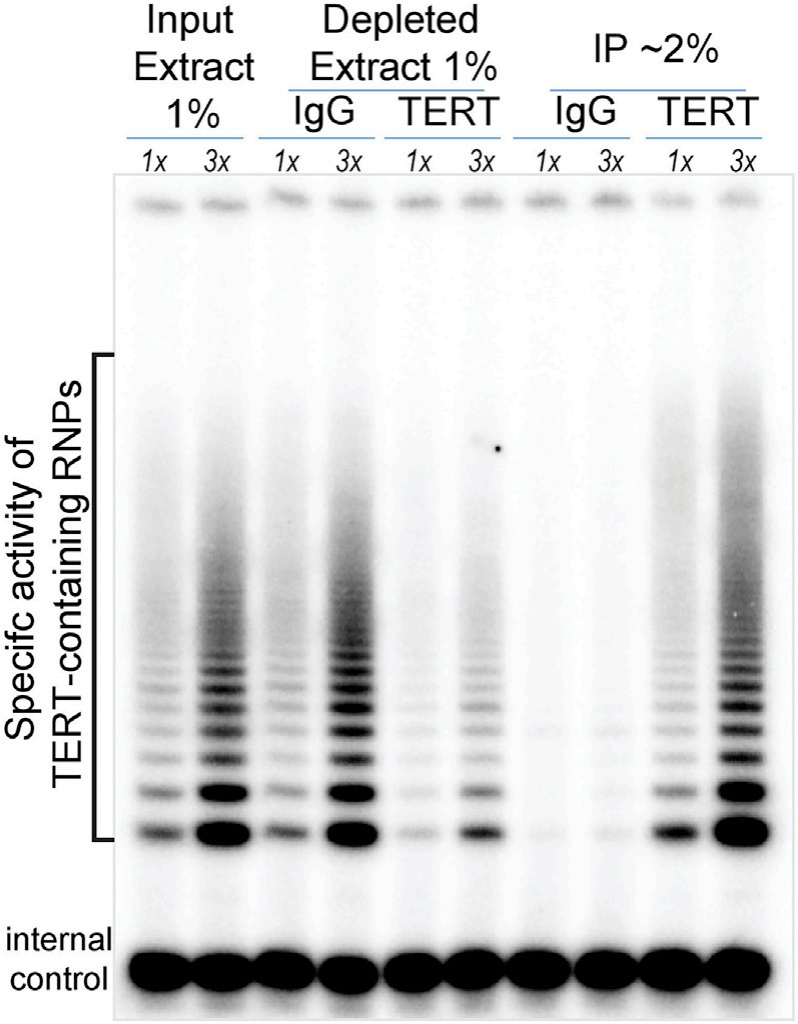
The anti-TERT antibody efficiently purifies and quantitatively depletes the endogenous telomerase RNP from nuclear extracts The ladder-like bands are Telomerase Repeated Amplification Protocol (TRAP) products- specific activity pattern of the telomerase RNP, serving as a surrogate to evaluate the presence of active telomerase RNP in each fraction tested (Major Step 4). The anti-TERT antibody （T421） was produced by immunizing a rabbit with the amino acid 421-493 of the human TERT protein fused with MBP, and affinity-purified using the GST-tagged antigen, as in Preparation 3-9. 1x and 3x denote the relative amount of each fraction assayed to demonstrate the linear range; 1x representing the corresponding percentage of total fraction volume as indicated for Input (1%), depleted (1%), and IP (2%).

3. Fractionate cells into a cytosolic extract “S100”, a high salt nuclear extract, and a DNase I-sensitive chromatin extract.
  ***Note:*** The procedure is modified from (Dignam et al., 1983), and was described in (Chen et al., 2014). For a large-scale extraction, we grow a minimum of 10 ml of Packed Cell Volume (PCV), an equivalent of ∼3 × 10^9^ cells. The large-scale ensures a sufficient amount of purified RNPs for multiple biochemical and structural readouts with necessary replicates, particularly helpful for low-expression RNPs, e.g., telomerase RNP - a couple of hundred assembled RNPs per HeLa cell (Xi and Cech, 2014).
  ***Alternatives:*** the original Dignam extraction has also been adopted at a smaller scale, with 3× 15 cm tissue culture plates ∼about 1 × 10^8^ mammalian cells (Folco et al., 2012). The smaller scale is sufficient to study more abundant RNPs, such as snRNPs with about 0.5 million copies per cell (Castle et al., 2010, Kiss and Filipowicz, 1992).
  **CRITICAL:** All tubes, homogenizers, and buffers should be pre-chilled before contacting cells and extracts. For all buffers, 1 mM DTT, 200 μM PMSF, RNase inhibitor, and a protease inhibitor cocktail should be added immediately upon usage. RNase-free grade reagents and practices should be used throughout.
  a. Harvest and wash cells with PBS, aspirate supernatant thoroughly, and measure the PCV (Figure 2E).
    **CRITICAL:** high viability (>95%) of starting cells is crucial for the procedure, as shown in the trypan blue-stained 293T cells (Figure 2A).

4. Affinity-purify endogenous RNPs
  **CRITICAL:** For all buffers used here, add 0.2 mM DTT (reduced to preserve IgG), 200 μM PMSF, RNase inhibitor, and a protease inhibitor cocktail immediately upon usage. RNase-free grade reagents and practices should be used throughout.
  ***Note:*** high-salt (420 – 450 mM) purification aims to reduce non-specific interactions, and to enrich salt-resistant, high-confidence components of RNP complexes.
  *Alternatives:* to preserve the salt-sensitive RNP components, dilute the nuclear extract 3-fold with 0.42× *Buffer C* (diluted in ddH_2_O) – the final NaCl will be ∼140 mM. Also, adjust NaCl to 140 mM in subsequent steps. Otherwise, proceed to the high salt purification as follows.
  a. Spinning down the input extract at 21,000 × *g* for 15 min at 4°C. Transfer the cleared supernatant to a new tube.
  b. Wash Protein A-conjugated agarose beads with PBS twice.
  c. Block the beads twice with *nuclear buffer420 w/ BSA* (see materials and equipment), each with 10min rotation at 4°C.
  d. Wash beads in *nuclear buffer420 w/o BSA* (see materials and equipment).
    ***Optional:*** a large batch of blocked and equilibrated beads can be stored as a 50% slurry in 4°C for up to 6 months.
  e. Preclear the extract with the prewashed Protein A beads by gently rotating at 4°C for 1 h.
    ***Note:*** per 1mL of extract, use 60 μL of prewashed protein A beads at 50% slurry.
  f. Pellet the beads at 7000 × *g* for 30 s at 4°C; transfer the supernatant to a new tube as the precleared nuclear extract.
  g. To affinity-purify RNP complexes from nuclear extracts, add 30 μg of purified anti-RBP antibody into 1mL of precleared nuclear extract.
  h. Gently rotate at 4°C for 60 min.
  i. Pipet 60 μL of the prewashed beads (50% slurry, from step 4-d) into the extract with an enlarged tip.
  j. Gently rotate at 4°C for no more than 4 h.
  k. Wash 4 times with ∼30 bead-volume of *Lys450 buffer* (see materials and equipment).
    ***Alternatives:*** to preserve salt-sensitive components of the RNP, wash instead with a milder *NP150 buffer* (see materials and equipment).
  l. Wash for an additional time with *1x direct assay buffer* (see materials and equipment).
  m. Resuspend the RNP-bound beads as a 50% slurry in *1x direct assay buffer w/ glycerol* (see materials and equipment). Snap freeze aliquots and store in −80°C freezer.
    ***Notes:*** set aside a small aliquot of the input, precleared, and depleted extracts, as well as immunoprecipitated fractions; analyze the RNP abundance in each by western and northern blotting or specific activity assays (see Figure 3 for an example).

***Alternatives:*** competitive elution of the bound RNP with 4× bed-volume of 0.6 μg/μL of the antigen peptide. Perform 5× sequential elutions at an ambient temperature around 22°C, 15min each. Pool peak fractions, which can be concentrated using a 10 kDa centrifugal filter device.

5. Biochemical reconstitution of purified RBP with RNPs.
  a. Express and purify recombinant RBP by affinity purification (See troubleshooting 3).
    ***Note:*** the genetically rescued CRISPR cell line generated in Major Step-2 serves as a cellular source for epitope-tagged RBP, which can be conveniently purified and eluted with commercially available reagents.
    **CRITICAL:** To facilitate the reconstitution of RBP with the corresponding RNA component of the RNP, it is helpful to vacant the RBP’s RNA binding site by removing endogenous RNA species that copurify with the RBP.
  b. To remove copurified RNAs, immobilize the RBP onto its corresponding affinity resin.
  c. Resuspend the resin in *Nuclear Buffer420 w/ BSA* containing 0.1 μg/μL RNase A, and gently rotate for 1 h at 4°C.
    ***Optional:*** the supernatant contains RNA-bridged protein partners of the RBP, and can be further analyzed by proteomic approaches.
  d. Wash the RBP-bound resin with a 100× bed-volume of *Lys450.*
    **CRITICAL:** it is imperative to thoroughly wash the resin to minimize the carryover of RNase to downstream applications that are RNase-sensitive, such as reconstitution, assaying RNP activity, and probing for RNP structure.
  e. Competitive elute the RBP from the resin by rotating in *1x direct assay buffer/ glycerol* containing 0.25 μg/μL peptide complementary to the affinity tag.
    ***Note:*** the presence of 30% glycerol helpsmaintain beads suspension during prolonged elutions.
  f. Combine the purified RBP with the RNPs at the desired molar ratio in *1x direct assay buffer w/ glycerol.*
    ***Note:*** both free and bead-bound fractions of RNPs can be used for the reconstitution.
  g. Incubate at 37°C for 30 min. Proceed to enzymatic assays or icSHAPE pipeline.

### Structural probing of purified RNPs by icSHAPE-seq

**Timing: 6 days**

*In vivo* icSHAPE-seq has been described in a step-by-step protocol (Flynn et al., 2016b), by which structural dynamics of the whole RNA transcriptome can be extrapolated from living cells. To gain insights into RBP binding *in vivo*, deproteinized, *in vitro*-folded RNA transcriptomes have been compared side-by-side to yield protein-independent, RNA-intrinsic structure profile (Spitale et al., 2015, Flynn et al., 2016a). However, the icSHAPE reactions inside living cells vs. test tubes are performed with different conditions and substrate complexity, potentially introducing experimental variations. Additionally, reliable extrapolation of full length, base-resolution structural profile of RNA demands sequencing depth and sufficient coverage , which can be challenging for noncoding RNAs and RNPs of low abundance.

**Figure 4:**
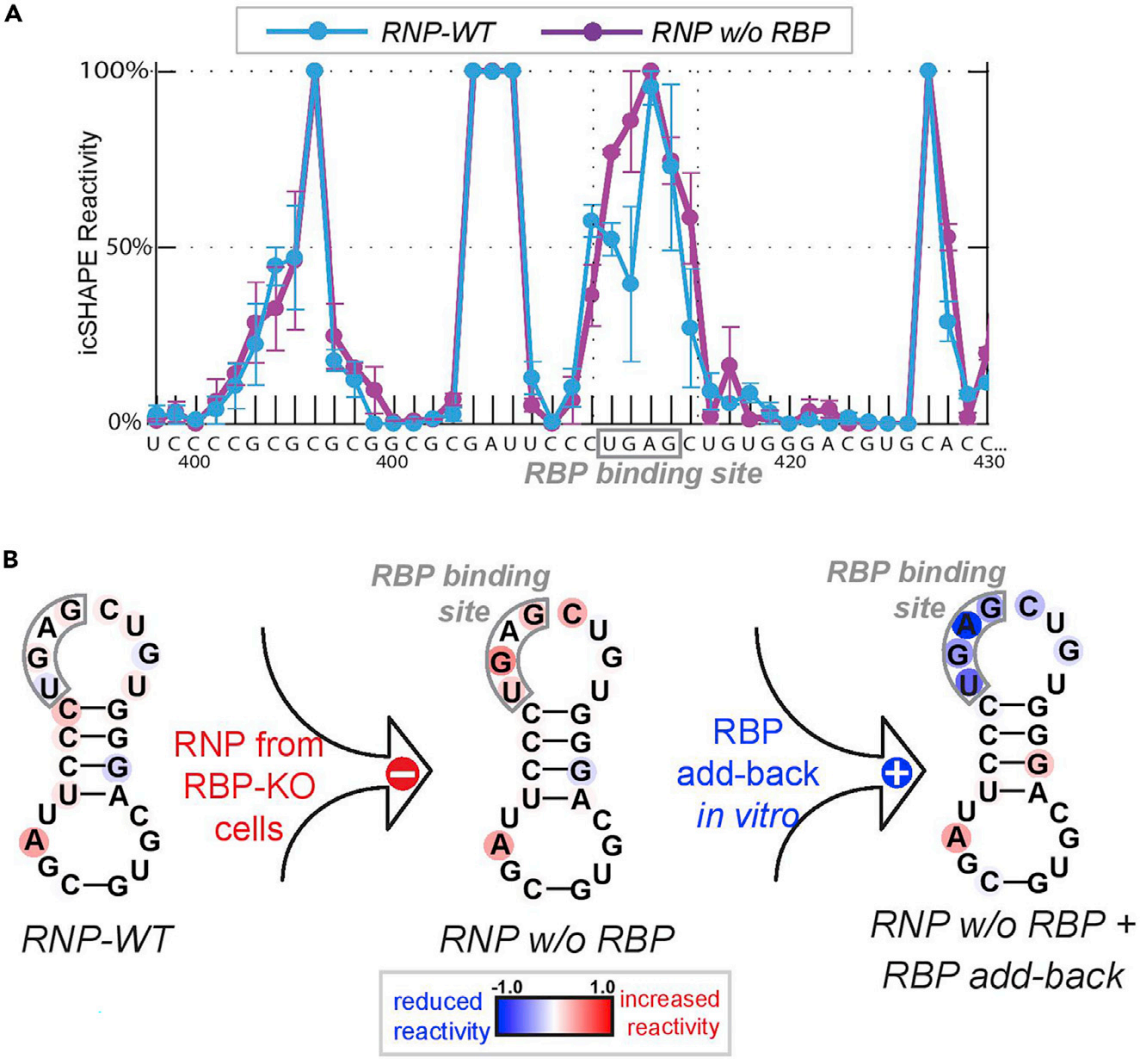
Pinpoint the structural footprint of a given RBP by comparing the icSHAPE reactivity of RNPs with or without the RBP (A) Raw icSHAPE reactivity, as a probability score (0%–100%), is graphed for RNA residues (298-430) of the telomerase RNA. The Blue track, telomerase RNP purified from WT cells; the Purple track, telomerase RNP purified from RBP-KO cell lines. RBP’s binding site is boxed in Gray. (B) Tracking the RBP footprint by RBP-KO and biochemical reconstitution. Relative icSHAPE reactivity from various RNP contexts is graphed onto an RNA secondary structure model. The RBP binding site “UGAG” is boxed in gray. Increased reactivity (shown in Red) at the UGAG is attributed to the loss of RBP from the RNP complex; decreased reactivity (shown in Blue) is caused by saturation of the binding site by adding back of excessive recombinant RBP *in vitro*, which assembled with the RNP purified from RBP-KO cells (RNP w/o RBP). The RBP’s additional distal binding sites at the P6.1 and P6b of telomerase RNA are not shown for simplicity. The differential icSHAPE reactivity profile (−1.0 to 1.0) from each context is derived by subtracting the value of an independent WT-RNP sample. Error bars represent the SD from two technical replicates.

To ensure high-quality structural profiling of candidate RNPs, we modified the icSHAPE protocol with the following considerations: firstly, to alleviate the complication related to depth and coverage, we enriched RNPs and RNAs of interest at a sufficient scale. Specifically, low abundant endogenous RNPs were affinity-purified from large-scale cultures of mammalian cells, and the corresponding RNA in naked form was synthesized at a high yield by *in vitro* transcription. Consequently, natively purified RNPs and their matching naked RNA can be normalized in terms of abundance and buffer condition, and be processed in parallel through the icSHAPE-seq pipeline (see the Graphic Abstract). Thirdly, to pinpoint the structural footprints of a given RBP in an unbiased fashion, the icSHAPE activity of RNPs with or without the RBP can be differentially compared (Figure 4). To further validate an observed structural alternation that depends on an RBP, recombinant RBP can be reconstituted at controlled stoichiometry with the RNP (Figure 4) or the naked RNA (data not shown). This discovery provides a structural framework to understand TCAB1’s contributions to catalysis and assembly of the telomerase RNP and attests to the unbiased power of icSHAPE in revealing structural dynamics within an RNP.

**Figure 5:**
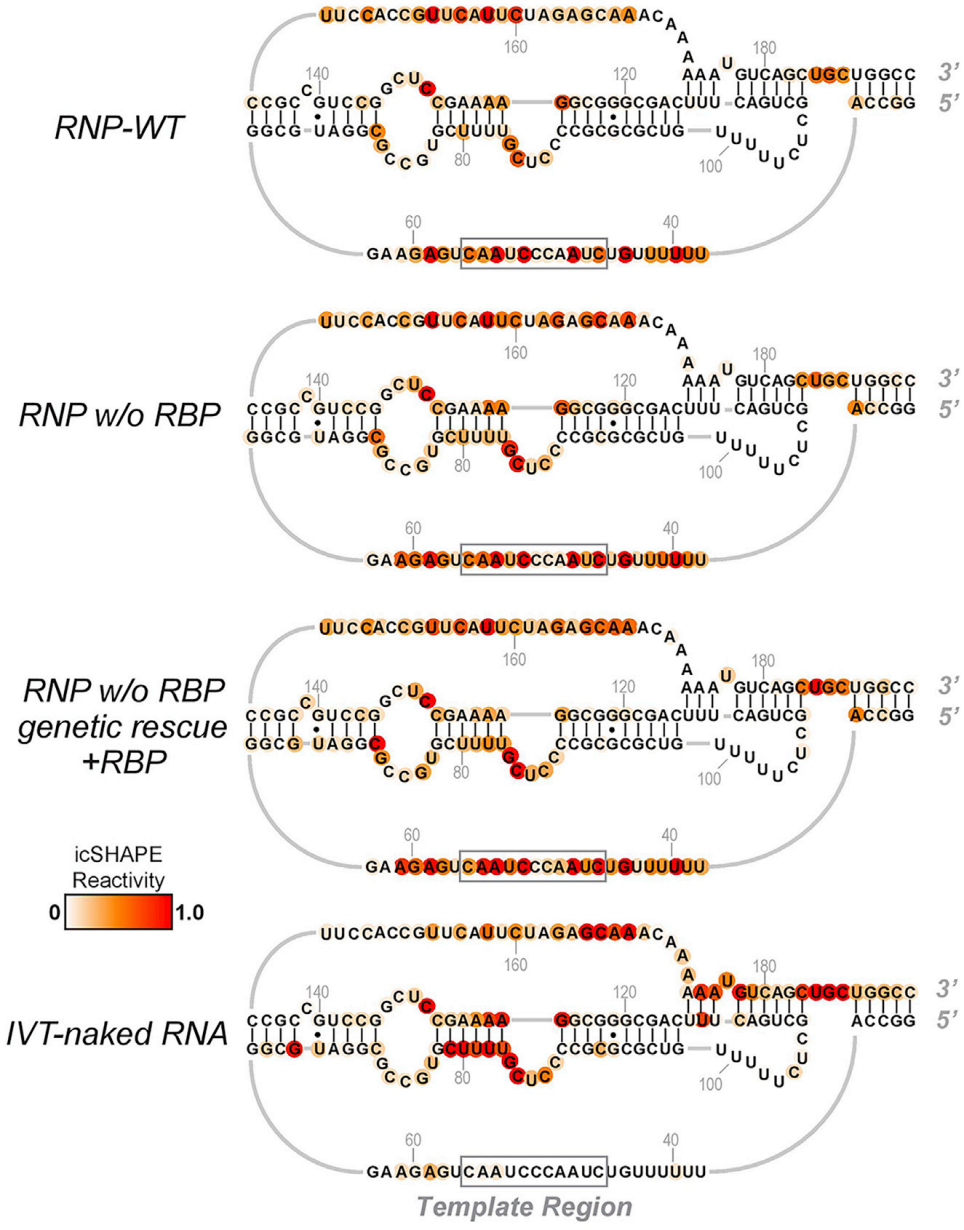
Differential icSHAPE profile of RNPs vs. naked RNA distinguishes RNA-intrinsic vs. RNP-specific structural features Raw icSHAPE reactivity profiles of three RNPs are compared with the naked RNA that is IVT-synthesized and *in vitro*-folded. Regardless of the RBP status, icSHAPE profiles are similar among RNPs, particularly the high reactivity at the template region (boxed in gray). In comparison, the naked IVT-RNA shows a reactivity profile largely similar to RNPs, except for the template region, showing diminished reactivity instead. This unbiased comparison suggests that the template region of the RNA, upon assembling into the RNP context, undergoes a structural transition from a buried and inaccessible state, to an open, solvent-accessible state, which has implications in substrate binding and RNP catalysis.

As a proof-of-concept, the biochemical reconstitution of TCAB1, a WD40-containing RBP, decreases the icSHAPE signal at the “UGAG” box – terminal loop residues of the telomerase RNA (Figure 4), indicating icSHAPE can accurately capture TCAB1’s footprint at the conserved CAB box important for telomerase localization in cells (Cristofari et al., 2007). Moreover, we revealed TCAB1’s distal structural footprints at P6.1 and P6b, two base-paired stem helices within the three-way-junction of the telomerase RNA. Lastly, structural dynamics specific to the RNP context can be uncovered by comparing and contrasting with the structure of the naked RNA (Figure 5), generated in Preparative steps 10–15.

6. Parallel icSHAPE modifications of purified RNPs and IVT-RNA by NAI-N_3_
  a. Normalize the molar concentration of complexed RNA and the IVT-RNA by northern blotting.
  b. Equilibrate WT-RNP, KO-RNP, and RNP + reconstituted RBP, and the IVT-RNA at 37°C for 30 min.
    **CRITICAL:** for accurate structural comparison, it is important to process relevant samples throughout the icSHAPE pipeline in parallel to minimize variations caused by the batch effect.
    ***Note:*** all fractions should be in the same buffer condition, such as in *1x direct assay buffer*.
  c. For each fraction, divide evenly into 4× reaction tubes.
    i.TUBE1: NAI-N_3_-treatment, replicate1
    ii.TUBE2: NAI-N_3_-treatment, replicate2
    iii.TUBE3: DMSO/mock-treatment, replicate1
    iv.TUBE4: DMSO/mock-treatment, replicate2
  d. Add 100 μM of NAI-N_3_ or an equal volume of DMSO to corresponding reaction tubes, incubate reactions at 37°C for 12 min in a thermomixer at 1000 rpm.
    **Pause point:** Stop reactions by addition of Trizol LS reagent.
    **CRITICAL:** the timing and the incubation condition across samples should be handled with precision and consistency.
  e. Precipitate total RNAs, and resuspend them in ddH_2_O.
  f. Resuspend RNAs in 2.5× volume of 100% ethanol, and purify RNAs using an RNA spin column.
  g. Quantitate the purified RNAs with the Qubit RNA HS assay and agarose gel.
    ***Note:*** 500 ng of purified RNAs are needed for successful library construction.

7. **cDNA library construction for NGS sequencing, and data analysis** according to (Flynn et al., 2016b)

***Note:*** Visualize icSHAPE reactivity at base-resolution onto an RNA secondary structure model (Figures 4 and 5), using the HiTRACE software package version 2.0 (Yoon et al., 2011).

## Expected outcomes

A successful nuclear extraction procedure is expected to solubilize native RNPs with high specific activity and low level of contaminated DNase and RNase. Key visuals are provided in Figure 2 as reference points for the extraction. In addition, IP-grade antibody – as instructed in the preparative step 2 – should be able to quantitatively enrich and deplete active RNP components and activity from the nuclear extract (Figure 3). This serves as desired QC indicators for the quality of the antibody, for high solubility of extracted RNPs, and for the low background contamination of the extract.

An icSHAPE-seq probing of endogenous telomerase RNPs with varying subunit composition is shown in Figure 4. It is expected that known RBP binding sites, e.g., the “CAB box” bound by TCAB1, within the RNP should exhibit differential icSHAPE-reactivity. It is also expected that additional RBP footprints can be captured, providing unbiased structural probing of the intra-RNP dynamics, e.g., two helices of the CR4/5 domain of telomerase RNA distant from the CAB box.

The icSHAPE profile of naked RNA synthesized *in vitro* provides a ground truth for RNA folding independent of RBPs and RNA PTMs. By comparing such with the very same endogenous RNA molecule –assembled with RBPs and decorated with PTMs, it is expected to reveal RNP-specific structural dynamics, such as the decompaction of the template region of telomerase RNA upon its assembly into the RNP context (Figure 5).

## Limitations

***Limitation1.*** Engineering a reliable knockout cell line is crucial to reveal an RBP’s structural and functional contributions to the RNP. However, for those RBP genes that are essential for cell growth, a clean knockout in cells is not feasible, following the CRISPR-KO approach described in Major Step-1. Instead of complete KO clones, hypomorphic clones with lowered target expression are often derived. Due to the heightened clonal variation and the residual RBP, analyzing multiple independent clones and genetic rescuing are recommended.

Moreover, we do note that some of those apparent CRISPR-KO clones in early passages can develop “escapers” that partially or fully restore the expression and function of the once inactivated gene. This has been often observed in clones where a single CRISPR indel disrupted the ATG or the coding frame at an early exon. Cellular adaptive strategies, including alternative start codon usage, internal ribosome entry, alternative splicing, and paralog complementation have been reported to generate hypomorphic or neomorphic alleles. Thus, carefully designed guide RNA pairs that flank the functionally important region, or even the whole gene locus should be considered to generate reliable loss-of-function cells (Canver et al., 2014, Bauer et al., 2015).

***Limitation2.*** We have observed a level of variations in icSHAPE measurements between biological replicates, and some considerable variations between experimental batches. These variations could be caused by differential amounts and complexities of the starting materials. In addition, the modification kinetics by NAI-N_3_ is sensitive to multiple parameters, including reaction time, temperature, substrate concentration, and complexity. Furthermore, the complexity and yield of the cDNA libraries between the NAI-N_3_ treatment and the DMSO-control groups become quite different after the biotin-selection step. Finally, the ensuing library amplification requires a different number of PCR cycles between the two groups, and the subsequent NGS needs differential depths to compensate for the lower yield of the NAI-N_3_ groups. These experimental variables could introduce challenges for quantitative assessment of icSHAPE reactivity at a given RNA base and across batches.

***Limitation3.*** A productive biochemical reconstitution provides direct evidence for RBP functions *in vitro* but depends on the availability of RBP preparations with sufficient quality and quantity, which are not always attainable. RBPs that are predominantly misfolded, aggregated, or copurified with contaminating activities that interfere with downstream biochemical readouts should not be used. Additionally, RBPs in certain cellular contexts may stably co-purify with (sub)stoichiometric endogenous factors, which would complicate the interpretation of the reconstitution result. Finally, certain RBP chaperons are responsible for proper folding and maturation of diverse RNA substrates (Yong et al., 2010, Zhang et al., 2008). The inactivation of such RBPs can lead to transcriptome-wide alternations that affect the specificity and efficiency of the antibody, thereby introducing variable background and complexity to the resulting RNP preparations.

## Troubleshooting

### Problem 1

A significant portion of the *in vitro*-transcribed RNA products, including sgRNA (Preparative-1), Cas9 mRNA, or naked RNA for icSHAPE (Preparative-10), are shorter than the expected full-length. See Figure 1 for the desired products and the “bad” outcome. This could be caused by RNase contamination or suboptimal conditions for the reverse transcription by T7 polymerase.

### Potential solution

To limit the action of potential contaminating RNases, besides the standard precautions working with RNase-free environment and reagents, we keep RNA-containing tubes on ice whenever possible, and immediately snap-freeze any un-used RNAs using dry ice or liquid nitrogen for storage in a −80°C freezer.

Secondly, prolonged incubation of T7 polymerase reactions in 37°C may increase the overall RNA yield, but negatively impact the RNA quality, potentially due to RNA degradation, enzyme exhaustion, and/or substrate depletion, which stalls the elongating RNA polymerase and results in the comet-tail pattern shown in Figure 1. To combat this, we found that shortening the reaction time has improved the yield of full-length RNA products significantly. To compensate for the shortened reaction time, we saturate IVT reactions with DNA templates to maximize the (re)initiation rate of T7 polymerase. To prepare for DNA templates by PCR or by linearization of a circular plasmid, we recommend optimizing the PCR condition or the restriction digestion to maximize the yield of the desired DNA template, so that no further gel purification is needed. This is critical because we have found that carryovers from agarose gel extraction can negatively impact IVT reactions.

### Problem 2

In Major step 3, the packed cell pellet is expected to swell in volume after the hypotonic incubation (Figures 2B and 2F). If not observed, a large fraction of cells may have ruptured cell membrane prematurely, which usually results in poor yield and quality of the final extracts.

### Potential solution

To reduce premature damages to the cell membrane, the starting cells should have viability higher than 95% before harvesting (Figure 2A). Secondly, we prefer to start the extraction protocol with freshly harvested cells, as both freezing/thawing and prolonged incubation on ice cause cell rupturing and clumping. Furthermore, cells, nuclei, and chromatin should be handled very gently, and be kept cold as much as possible.

### Problem 3

As shown in Figure 2G and Major step 3-p, a successful nuclear extraction should yield a transparent supernatant without visible debris. The appearance of debris in the extract can be caused by unwanted shearing/releasing of DNA and chromatin, which generally correlates with a higher level of contaminating nuclease and protease activities in the extract.

### Potential solution

It is important to avoid exposing nuclei suspension to high NaCl concentration from the 5M stock during Major step 3-l. As instructed in Methods Video S1, we apply a slow but steady drop-wise stream of the 5M NaCl stock solution into the nuclei, while maintaining a gentle and efficient swirling to mix.

### Problem 4

In Major step5, RBP add-back may not reconstitute structural or functional activities of interest.

### Potential solution

To test the contribution of an RBP preparation to a given biochemical readout, we recommend titrating the RBP over a wide range of molar concentrations. To produce active RBPs for reconstitution, it is recommended to test different expression systems (prokaryotic vs. baculovirus vs. mammalian cell hosts). Also, different buffer usage, tagging, and purification strategies, e.g., the RNase pre-treatment in Major step 5. Furthermore, introducing mutations at the functional domain(s) of recombinant RBPs would help pinpoint the specific RBP property that contributes to any reconstituted activity.

### Problem 5

In Major step 6, variations in icSHAPE profiles among biological replicates and assay batches.

### Potential solution

To lower variations and batch effect, we recommend users handle relevant samples in parallel throughout the icSHAPE pipeline. Moreover, the starting cell numbers for RNA extraction or RNP purification should be kept at a comparable scale. Furthermore, RNAs/RNPs of a similar molar concentration, buffer condition, and complexity should be icSHAPE-treated and mixed using a multichannel pipet and processed with strict consistency across samples.

## Resource availability

### Lead contact

Further information and requests for resources and reagents should be directed to and will be fulfilled by the lead contact, Steven E Artandi (sartandi@stanford.edu)

### Materials availability

Plasmids generated in this study are available through Addgene (Addgene IDs listed in the key resources table)

### Data and code availability

The icSHAPE datasets generated during this study are available at NCBI’s Gene Expression Omnibus, through the GEO Accession number: GSE97486 (https://www.ncbi.nlm.nih.gov/geo/query/acc.cgi?acc=GSE97486)
